# Discussing the safety and effectiveness of transcatheter arterial embolization combined with intravenous chemotherapy in treating locally advanced breast cancer

**DOI:** 10.1038/s41598-024-56642-w

**Published:** 2024-03-12

**Authors:** Jingjun Zhang, Jie Zhang, Xiangke Niu, Yongxiang Zhou, Yufeng Guo, Yuanzhi Wang, Feng Shou

**Affiliations:** 1 Jianyang People’s Hospital, Jianyang City, 641400 Sichuan Province China; 2 The First Affiliated Hospital of Chengdu Medical College, Chengdu City, Sichuan Province China; 3 Affiliated Hospital of Chengdu University, Chengdu City, Sichuan Province China

**Keywords:** Locally advanced breast cancer (LABC), Neoadjuvant chemotherapy, Drug-eluting beads, Safety evaluation, Cancer, Medical research, Oncology

## Abstract

To investigate the efficacy and safety of drug-eluting bead-transarterial chemoembolization (DEB-TACE) combined with systemic chemotherapy in HR+/Her2− locally advanced breast cancer (LABC) patients. A controlled study was conducted on LABC patients treated at Jianyang People’s Hospital and the First Affiliated Hospital of Chengdu Medical College from December 2020 to June 2022. The patients were randomly divided into the experimental group and the control group. The experimental group received DEB-TACE combined with the TAC regimen (175 mg/m^2^ paclitaxel-loaded albumin, 50 mg/m^2^ Doxorubicin, and 500 mg/m^2^ cyclophosphamide), while the control group received the TAC regimen intravenously. The therapeutic efficacy was evaluated using the mRECIST criteria. Statistical analysis was performed using SPSS 22.0 software, and baseline characteristics, overall response rate (ORR), pathological complete response (PCR), adverse reactions, and complications were compared between the two groups using paired t-test and chi-square test. A total of 60 patients were included, with 30 patients in the experimental group (50%) and 30 patients in the control group (50%). After the first treatment, the ORR was 90% in the experimental group and 60% in the control group (*P* < 0.05). The overall ORR was 100% in the experimental group and 83% in the control group (*P* < 0.05). PCR was achieved in 14 patients (47%) in the experimental group and 4 patients (13%) in the control group. The main adverse reactions in the experimental group were skin blistering, pigmentation, and pain. There was no statistically significant difference in vomiting and grade II or above bone marrow suppression between the two groups. No grade III or above adverse events occurred in either group. The comparison of tumor shrinkage between the two groups was *P* = 0.051, and axillary lymph node shrinkage was *P* < 0.05. The use of drug-eluting beads in combination with neoadjuvant chemotherapy is a feasible and safe treatment option for locally advanced breast cancer patients.

## Introduction

Data analysis from the National Cancer Center (NCC) of China in 2022 revealed that breast cancer has become the most common and deadliest malignancy among women^1^. Research has shown that neoadjuvant chemotherapy (NAC) has become an important approach for treating locally advanced breast cancer (LABC)^2–4^. NAC was first employed in LABC patients in 1970. With a deeper understanding of the biological behavior of breast cancer and the evolution of treatment concepts, NAC has become an integral part of systemic therapy for breast cancer, especially in reducing the size of primary tumors and the spread to axillary lymph nodes. The CTNeoBC meta-analysis demonstrated that breast cancer patients who achieved pathological complete response (pCR) after NAC had better prognosis compared to those who did not achieve pCR^5^.

Unfortunately, there are no targeted agents available for neoadjuvant treatment of HR+/Her2− LABC patients. Traditional intravenous chemotherapy with taxanes and anthracyclines achieves only a complete pathological response (pCR) rate of 6–12%, and 5–10% of patients experience tumor progression during neoadjuvant chemotherapy^6–8^. Therefore, we propose using CalliSpheres® drug-eluting beads transarterial chemoembolization (DEB-TACE) in combination with the TAC regimen during neoadjuvant chemotherapy for LABC patients.

DCB-TACE, using drug-eluting beads for arterial embolization, can slowly release chemotherapeutic agents to kill tumor cells in the embolized tumor region. It has been widely used in liver cancer and liver metastases, with its safety and efficacy well validated^9–12^. However, the use of DEB-TACE in LABC to improve overall response rate (ORR) and achieve pCR has been rarely reported in the literature. Here, we aim to summarize the recent effects of using drug-eluting beads arterial embolization (DCB-TACE) for LABC and provide more personalized treatment options for LABC patients based on the research findings.

## General information

### Inclusion and exclusion criteria

Inclusion criteria for this study were: 1. Age 18–75 years, any gender; 2. Pathologically confirmed newly diagnosed locally advanced breast cancer; 3. Eastern Cooperative Oncology Group (ECOG) performance status ≤ 2; 4. HR+/Her2− status; 5. Negative pregnancy test for premenopausal women and agreement to use effective contraception during treatment and for 1 year afterward.

Locally advanced breast cancer can be defined in three situations: the primary lesion diameter is larger than 5 cm, the tumor is adherent and fixed to the skin or chest wall, and the regional metastatic lymph nodes are fused with each other. This includes T(0–2)N2M0, T3N(1)M0, T4 any NM0, and any TN3M0.

Exclusion criteria were: 1. Her2+ status; 2. Poor coagulation function with INR > 1.5, or currently receiving anticoagulation therapy or known bleeding disorders; 3. Unstable angina or recent myocardial infarction (within one year); 4. Recent infection requiring antibiotic treatment; 5. White blood cell count < 3000 cells/mm^3^ or platelet count < 50,000/mm^3^ without splenomegaly; 6. Renal insufficiency (creatinine > 2 mg/L); 7. AST and/or ALT levels > 3 times the upper limit of normal; 8. Concomitant conditions or social environment that would prevent compliance with the study protocol or jeopardize patient safety; 9. Patients with other primary tumors concurrently.

A total of 88 LABC patients treated at Jianyang People’s Hospital and the First Affiliated Hospital of Chengdu Medical College from December 2020 to June 2022 were enrolled and randomly divided into the experimental group (n = 30) and the control group (n = 30) after excluding patients with Her2+ (n = 9), distant metastasis (n = 5), HR−/Her2− (n = 8), unstable angina or recent myocardial infarction within one year (n = 3), and renal dysfunction (n = 3). All patients were females with pathologically confirmed invasive ductal carcinoma with axillary lymph node metastasis.

This study was conducted in accordance with the WMA Declaration of Helsinki and the CIOMS International Ethical Guidelines for Biomedical Research. The study was approved by the Medical Ethics Committee of Jianyang People's Hospital (JY202012), and the trial registration number is ChiCTR2000041001.

### Technical protocol

The experimental group underwent Seldinger technique for femoral arterial puncture, followed by angiography of the subclavian artery to selectively perfuse the tumor-feeding vessels with chemotherapy agents-loaded microspheres. One session of DEB-TACE was performed, and after the first treatment, the extent of tumor necrosis was evaluated using breast enhanced MRI to analyze the treatment response rate and adverse reactions. Repeat treatment was performed after every 2 cycles of the TAC regimen, and a biopsy was conducted after achieving complete remission by modified Response Evaluation Criteria in Solid Tumors (mRECIST) criteria. DEB-TACE was stopped after confirming pathological complete response (PCR), and subsequent chemotherapy was completed. Modified radical mastectomy for breast cancer was performed if indicated. The control group received systemic chemotherapy with the TAC regimen via intravenous infusion.

**Figure 1:**
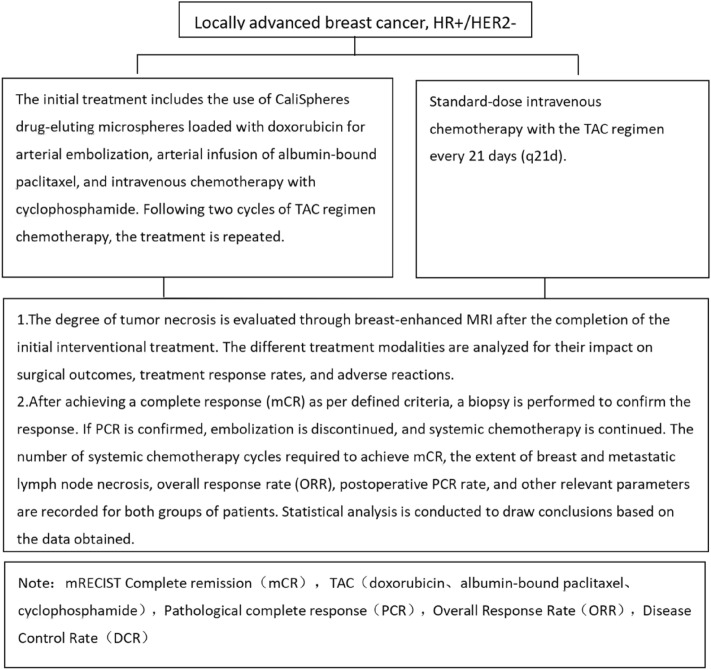
Research workflow diagram.

TAC regimen: Albumin-bound paclitaxel 175 mg/m^2^, Doxorubicin50 mg/m^2^, cyclophosphamide 500 mg/m^2^ (See Fig. 1 for the technical route).

### Equipment and materials

The interventional procedure was performed using the Discovery IGS 730 system (GE, USA). CalliSpheres microspheres were produced by Suzhou Hengruijiali Bio-Engineering Technology Co., Ltd., China (Medical Device Production License: Jiangsu Food and Drug Administration Medical Device Production License No. 20090041; Product Registration Certificate No.: National Registration Certificate No. 20153771072; Product Standard Label: TZB/国4586-2013). The RAPIDTHRU microcatheter and guidewire kit was provided by Suzhou Hengruidi Medical Technology Co., Ltd., China (Medical Device Production License: Jiangsu Food and Drug Administration Medical Device Production License No. 20180050; Product Registration Certificate No.: National Registration Certificate No. 20183770117; Product Standard Label: J0001020001001).

### Preparation of drug-eluting beads

CalliSpheres drug-eluting beads (300–500 μm) were extracted into a 20 ml syringe and left for 2 min, after which the supernatant was expelled. Doxorubicin(50 mg/m^2^) was dissolved in 5 ml of 5% glucose solution based on the patient’s body surface area, and the solution was injected into the syringe containing CalliSpheres. The syringe was gently agitated to ensure thorough and uniform mixing. After 3 min of standing, the syringe was vigorously shaken for a total standing time of 20 min. The supernatant was aspirated and filtered through a 3um membrane into a 10 ml syringe for later use. Finally, the prepared CalliSpheres drug-eluting beads were mixed with non-ionic contrast agent in a 1:1 ratio.

### Interventional procedure

**Figure 2:**
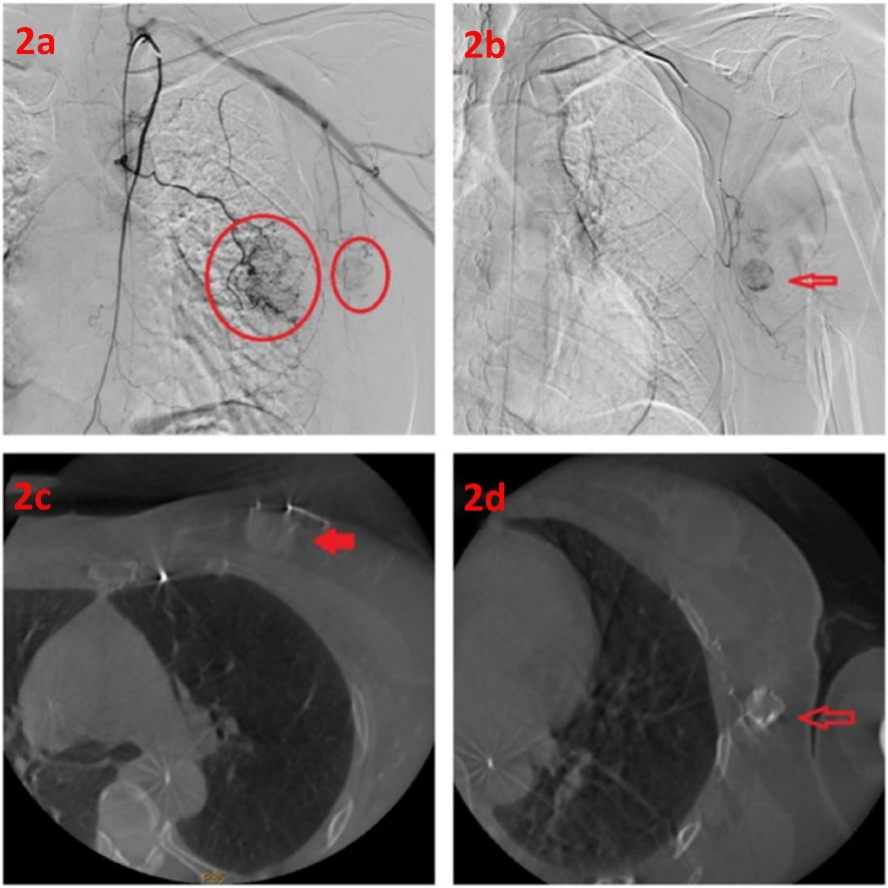
Intraoperative contrast imaging and CBCT imaging. (**a**), (**b**) Angiography of breast tumors and axillary metastatic lymph nodes was performed using microcatheter superselective intubation. (**c**), (**d**) CBCT scanning can visualize breast tumors and axillary lymph node metastasis.

**Figure 3:**
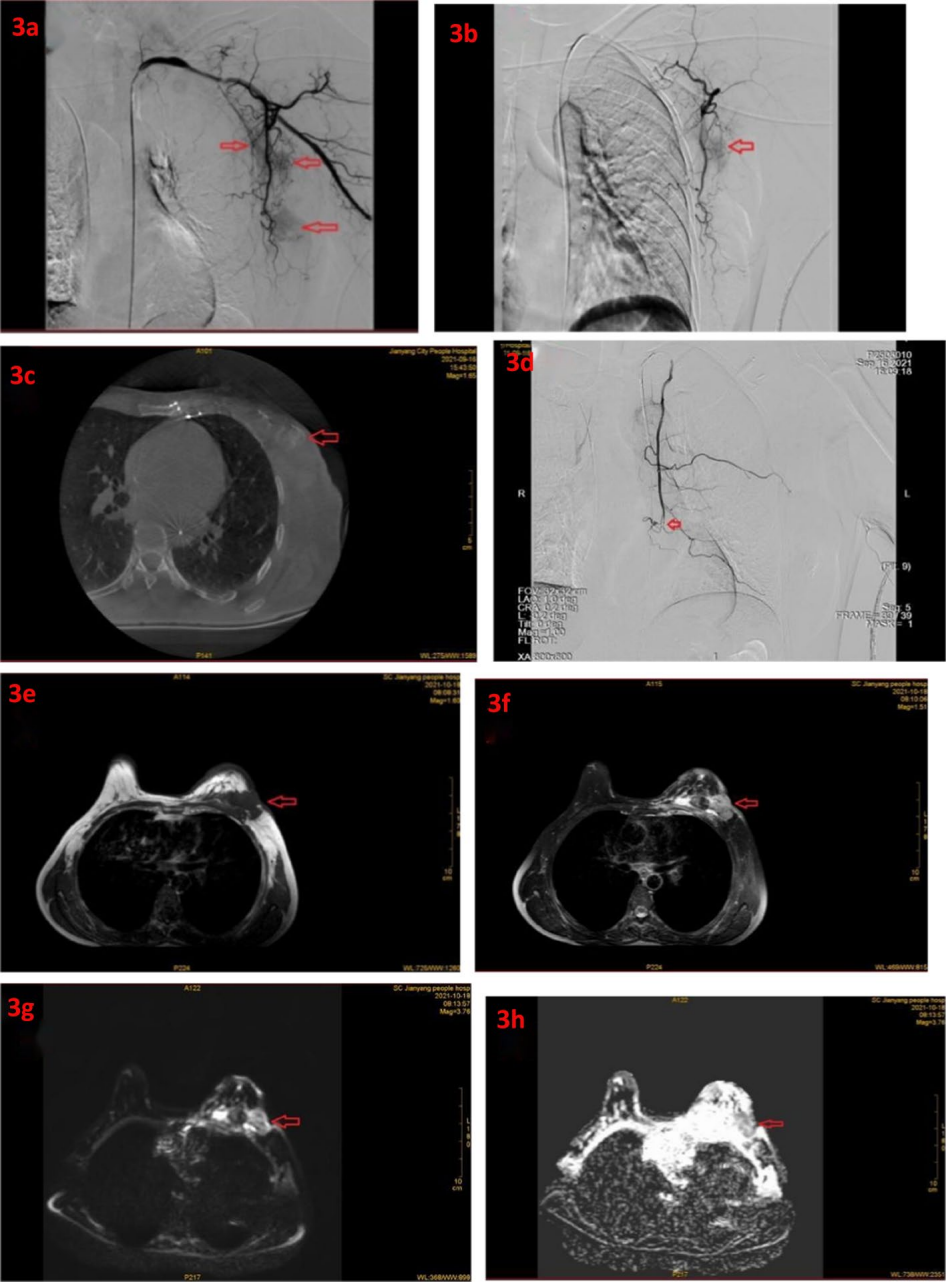
(**a**), (**b**) Intraoperative dyeing of breast tumors and axillary lymph nodes with metastasis; (**c**) Under CBCT, breast tumors are displayed; (**d**) Post-embolization contrast-enhanced images, with an arrow indicating a metallic coil; (**e**) T1-weighted imaging (T1WI); (f) T2-weighted imaging (T2WI); (**g**) Diffusion-weighted imaging(DWI); (**h**) Apparentdiffusioncoefficient (ADC).

The skin over the breast tumor and axillary lymph node metastases were protected with ice packs. The bilateral inguinal and perineal regions were disinfected, and the femoral artery was accessed using the Seldinger technique. A 5F diagnostic catheter (Cordis, USA) was placed at the opening of the subclavian artery. Digital subtraction angiography (DSA) was performed using the non-ionic contrast agent iopromide (Visipaque, GE Healthcare AS) at a flow rate of 3 mL/s and a total volume of 15 mL, with a pressure of 300 psi. Cone-beam CT (CBCT) scanning and three-dimensional image reconstruction with vessel tracking technology were used to identify the tumor-feeding vessels (Fig. 2). A 2.7F microcatheter (RAPIDTHRU microcatheter and guidewire system, China) was coaxially advanced to the tumor-feeding arteries. Spring coils (COOK, USA) were implanted distally in the non-tumor supplying arteries as protective embolization. After confirming satisfactory catheter position, albumin-bound paclitaxel (175 mg/m^2^) and filtered Doxorubicin solution were infused intra-arterially. All drugs were administered through a microinfusion pump, with albumin-bound paclitaxel infusion time being 20 min, and the filtered doxorubicin solution diluted to 50 ml with an infusion time of 30 min.The tumor-feeding arteries of the breast tumor and axillary lymph node metastases were perfused with half of the total drug dosage each. During the infusion, blood pressure cuffs were used to prevent drug reflux into the upper-arm vessels. The prepared CalliSpheres drug-eluting beads were embolized into the tumor-feeding arteries using the microcatheter. If the tumor blood flow slowed down or stagnated, the embolization was paused, and after 5 min, the embolization end-point was assessed. If the end-point was not reached, additional embolization was performed until tumor blood flow slowed down or stagnated (Fig. 3 shows the operative procedure and preoperative MRI imaging findings).

### Postoperative management

On the day of DEB-TACE, patients in the experimental group received intravenous cyclophosphamide (500 mg/m^2^) to supplement the dose of the TAC regimen. Skin cooling protection with ice packs over the embolized area was performed for 3–5 days. Changes in white blood cell count, BNP, CK-MB, and electrocardiogram were monitored. Pain relief was provided if the visual analog scale (VAS) score was > 5.

### Tumor Response

Tumor response was assessed by comparing pre- and post-treatment breast enhanced MRI images. The tumor response was classified as complete response (CR), partial response (PR), stable disease (SD), or progressive disease (PD) according to modified Response Evaluation Criteria in Solid Tumors (mRECIST) criteria^13^. Objective response rate (ORR), disease control rate (DCR), and PCR were calculated. Adverse reactions and complications were recorded and managed.

### Statistical analysis

Statistical analysis was performed using SPSS 22.0 software. Continuous variables were presented as means ± standard deviations. Paired t-tests were used to compare lesion changes between the two groups. Chi-square tests were used to compare efficacy and adverse reactions. A significance level of α = 0.05 and *P* < 0.05 were considered statistically significant.

### Ethical approval

This study was conducted in accordance with the WMA Declaration of Helsinki and the CIOMS International Ethical Guidelines for Biomedical Research. The study was approved by the Medical Ethics Committee of Jianyang People’s Hospital (JY202012).

### Informed consent

This study involves human participants (including the use of tissue samples), and we hereby confirm that informed consent has been obtained from all subjects and/or their legal guardians. Prior to the start of the study, detailed research explanations were provided to the participants or their legal guardians, and the purpose, procedures, and potential risks of their participation were clearly explained. They were given sufficient time to read and understand the relevant information and were encouraged to raise any questions. The participants or their legal guardians voluntarily chose to participate in this study after fully comprehending the research content, benefits, and potential risks.

Furthermore, we explicitly informed the participants or their legal guardians about their rights in participating in the study, including the freedom to withdraw from the study at any time without facing any adverse consequences, as well as the right to request the deletion or withdrawal of their data at any stage.

We have taken appropriate measures to protect the privacy and data security of the participants and will only use the data for research purposes. In the reporting of the results, we will ensure anonymization of personal identities and utilize aggregated data for analysis and presentation.

## Results

### Baseline characteristics of patients

A total of 60 patients were included in the study (Table 1), with 30 patients in the experimental group (50%) and 30 patients in the control group (50%). The average length of hospital stay was 6.5 days in the experimental group and 4.1 days in the control group. The average follow-up time was 10.8 months, and no tumor distant metastasis occurred. There were no statistically significant differences between the two groups in terms of average age, physical fitness score, menopausal status, hormone expression type, size of primary lesion and axillary lymph node metastasis, and associated medical conditions (*P* > 0.05).

**Table 1:** Clinical characteristics of study population.

Clinical features	Experimental group (n = 30)	Control group (n = 30)	t/χ2	*P*-value
Age (years, x ± s)	55.3 ± 5.7	57.5 ± 5.5		
PS score (%)				
0	21(70)	21(70)	0.300	0.584
1	9(30)	11(37)		
Postmenopausal (%)	23(77)	22(73)	0.089	0.766
Premenopausal (%)	7(23)	8(27)		
ER+, PR+	12(40)	15(50)	0.622	0.733
ER+, PR−	8(27)	7(23)		
ER−, PR+	10(33)	8(27)		
Primary lesion > 5 cm	18(60)	19(63)	0.079	0.791
Primary lesion ≤ 5 cm, lymph node involvement (%)	12(40)	11(37)		
Comorbidities (%)				
Yes	8(27)	7(23)	0.089	0.766
No	22(73)	23(77)		

### Evaluation of treatment efficacy

**Figure 4:**
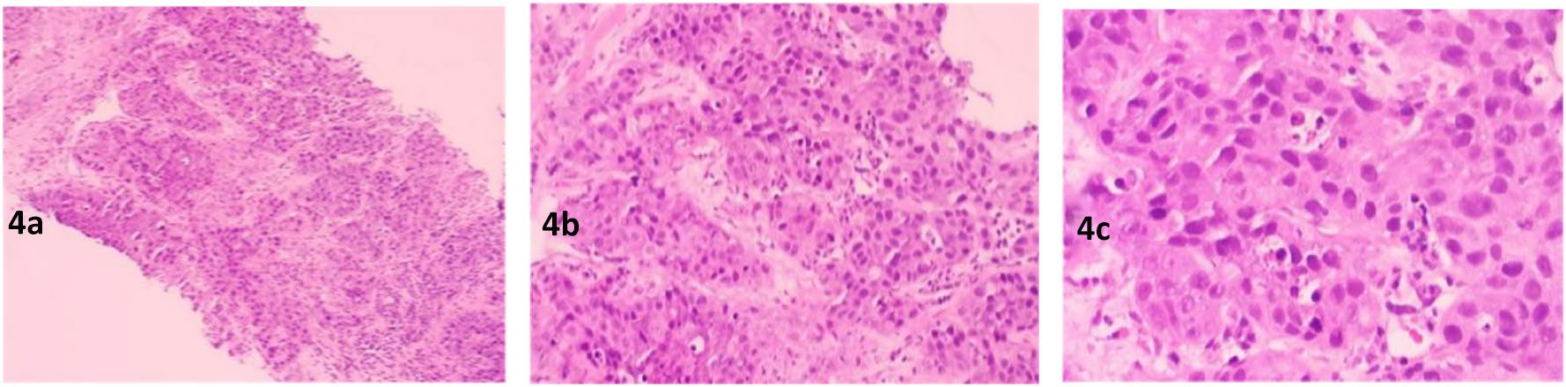
Pre-treatment. (**a**) The tumor cells exhibit a clustered or nest-like infiltrative growth pattern, HEX10. (**b**) The tumor cells show significant nuclear pleomorphism, with focal necrosis visible, HEX20. (**c**) The tumor cells have prominent nucleoli, with the presence of pathological mitotic figures easily observed, HEX40.

**Figure 5:**
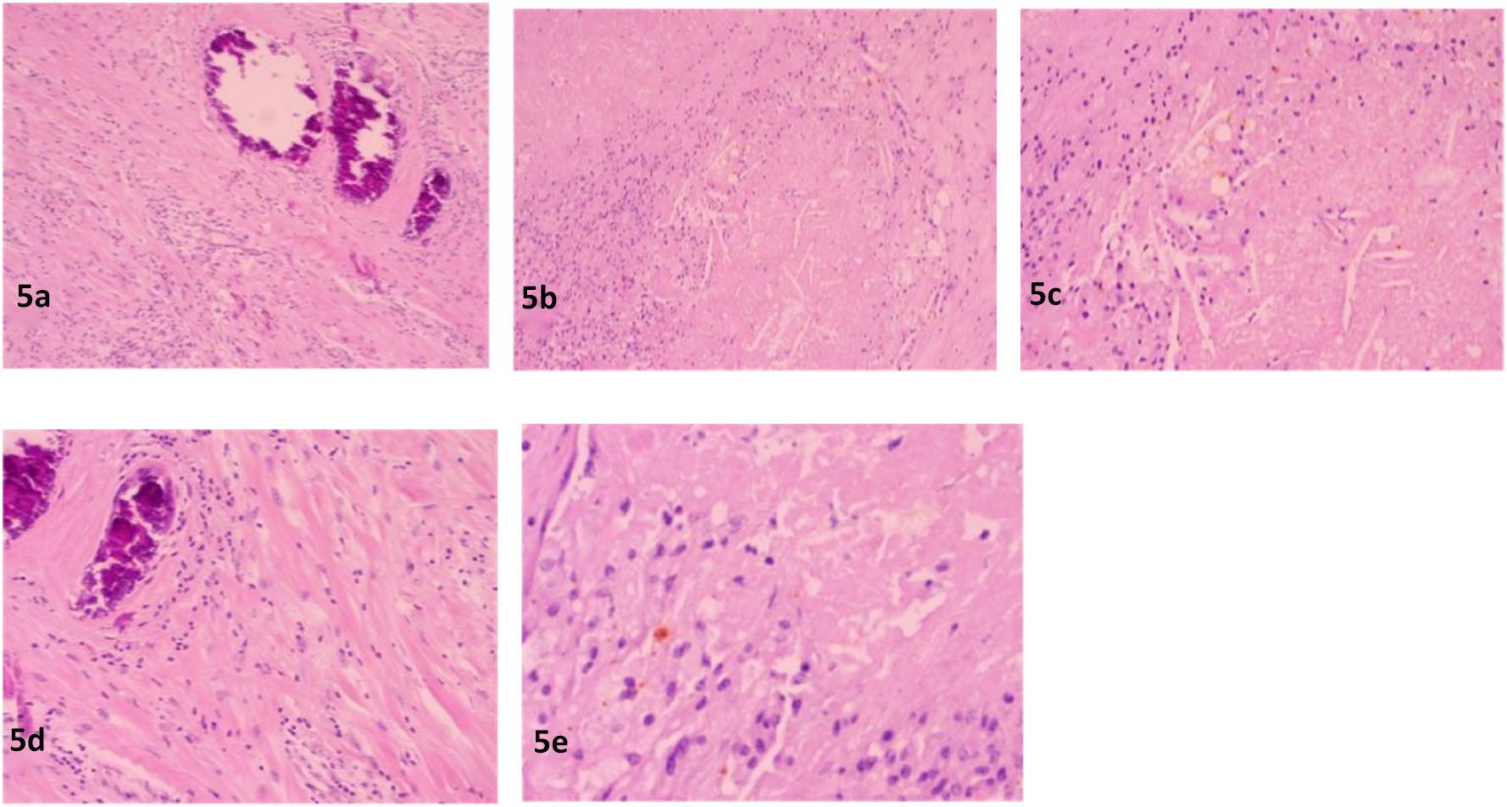
Post-treatment. (**a**) Fibrous tissue hyperplasia with infiltration of lymphocytes and focal calcification formation, HEX10. (**b**) Foamy tissue cell aggregation with coagulative necrotic foci formation, HEX10. (**c**) Foamy tissue cell aggregation with coagulative necrotic foci and cholesterol crystal formation, HEX20. (**d**) Fibrous tissue proliferation accompanied by infiltration of lymphocytes and focal calcification formation, HEX20. (**e**) Foamy tissue cell aggregation accompanied by coagulative necrotic foci formation, HEX40.

In the experimental group, a total of 56 sessions of drug-eluting bead transarterial chemoembolization (DEB-TACE) and 122 sessions of TAC regimen intravenous chemotherapy were completed. In the control group, a total of 224 sessions of TAC regimen intravenous chemotherapy were completed. The comparison of objective response rate (ORR) after the first DEB-TACE in the experimental group and the first chemotherapy in the control group showed a ratio of 90%:60% (*P* < 0.05). The overall ORR comparison between the experimental group and the control group showed a ratio of 100%:83% (*P* < 0.05) (Table 2). There were 14 patients in the experimental group who achieved pathologic complete response (PCR) (47%) without disease progression (Figs. 4 and 5 represent the histopathological changes before and after treatment), while 4 patients in the control group achieved PCR (13%). Three patients in the control group experienced disease progression but without distant metastasis, and 2 patients in the control group evaluated disease stability after 4 cycles of chemotherapy. These 5 patients underwent surgery earlier (Table 3). The comparison of disappearance of primary breast tumor between the two groups showed a *P* value of 0.051, and the disappearance of axillary lymph node metastasis showed a *P* value less than 0.05 (Tables 4, 5).

**Table 2:** Comparison of efficacy after first-line treatment in HR+/HER2−negative experimental group and control group (%).

	Number	CR	PR	SD	PD	ORR	DCR
Experimental group	30	0(0)	27(90)	3(10)	0(0)	27(90)	30(100)
Control group	30	0(0)	18(60)	12(40)	0(0)	18(60)	30(100)
χ2-value		–	7.200	7.200	–	7.200	–
*P*-value		–	0.007	0.007	–	0.007	–

**Table 3:** Comparison of overall efficacy between the experimental group and the control group in HR+/HER-2 negative patients (%).

	Number	CR	PR	SD	PD	ORR	DCR	PCR
Experimental group	30	14(47)	16(53)	0(0)	0(0)	30(100)	30(100)	14(47)
Control group	30	4(13)	21(70)	2(7)	3(10)	25(83)	27(90)	4(13)
χ2-value		7.933	1.763	2.069	3.158	5.455	3.158	7.937
*P*-value		0.005	0.184	0.150	0.076	0.020	0.076	0.005

**Table 4:** Comparison of overall tumor regression between the two groups.

Tumor location	Experimental group		Control group	
	Pre-treatment	Post-treatment	Post-treatment	Post-treatment
Primary tumor	4.98 ± 1.02	0.82 ± 086	5.13 ± 0.86	1.63 ± 0.89
t-value	18.393		17.867	
*P*-value	0.000		0.000	
Lymph nodes	2.55 ± 0.66	0.35 ± 0.46	2.31 ± 0.56	0.73 ± 0.49
t-value	15.666		11.507	
*P*-value	0.000		0.000

**Table 5:** Comparison of primary lesion and metastatic lymph node grouping.

	Experimental group variations	Control group variations
Degree of changes in primary lesions before and after treatment in two groups	4.16 ± 1.24	3.50 ± 1.07
t-value	2.036	
*P*-value	0.051	
Degree of changes in lymph nodes before and after treatment in two groups	2.20 ± 0.77	1.58 ± 0.75
t-value	3.250	
*P*-value	0.003	

### Comparison of adverse reactions

The main adverse reactions in the experimental group were skin blisters, pigmentation, and pain after DEB-TACE. The visual analog scale (VAS) score ranged from 3 to 5, with one patient having a VAS score greater than 7, requiring the use of morphine for pain management. One patient in the experimental group developed infected skin blisters leading to breast abscess. There were no statistically significant differences between the two groups in terms of vomiting and Grade II or higher bone marrow suppression, and no Grade III or higher adverse events occurred (Table 6).

**Table 6:** Comparison of treatment-related adverse reactions between the experimental group and the control group in patients (%).

	Experimental group	Control group	χ2-value	*P*-value
Number of subjects recruited	30	30		
Blister	8(27)	0(0)	9.231	0.002
Breast abscess	1(3)	0(0)	1.017	0.313
Pigmentation	8(27)	0(0)	9.231	0.002
Vomiting	6(20)	5(17)	0.111	0.739
Grade II or above	2(7)	3(10)	0.218	0.640
Pain	5(17)	0(0)	5.455	0.020
Delayed wound healing	0(0)	0(0)	–	–

## Discussion

HR+/HER2− (hormone receptor-positive/human epidermal growth factor receptor 2-negative) breast cancer is the most common subtype of breast cancer, accounting for approximately 70% of all cases in women. It is associated with prognosis in breast cancer patients, making its treatment crucial. Locally advanced breast cancer (LABC) is a subset of breast cancer characterized by late-stage local involvement without distant metastasis, accounting for approximately 10% of female breast cancer cases. Neoadjuvant chemotherapy is a common treatment approach for LABC as it effectively reduces or eliminates micrometastases and inhibits tumor cell activity, thus reducing the possibility of distant metastasis after surgery^14–25^. It also allows for the assessment of tumor chemosensitivity, which is beneficial for adjuvant chemotherapy after surgery. Unfortunately, the proportion of LABC patients with HR+/HER2− breast cancer achieving pathologic complete response (pCR) after neoadjuvant chemotherapy is low, especially in patients with estrogen receptor-positive (ER+) or progesterone receptor-positive (PR+) tumors who are less sensitive to chemotherapy. With the discovery of estrogen receptor and other signaling pathway crosstalk, as well as the emergence of novel targeted drugs, combination therapy with targeted agents and endocrine therapy has significantly improved treatment efficacy. A study on endocrine therapy for advanced postmenopausal breast cancer achieved a clinical breakthrough in solid tumors with histone deacetylase (HDAC) inhibitors for the first time worldwide^16^. This represents a new direction of effort, but pCR rates remain low, and there is still a long way to go for endocrine neoadjuvant therapy.

Currently, chemotherapy remains the standard treatment for locally advanced HR+/HER2− breast cancer, and improving drug utilization is crucial for improving treatment outcomes. In addition to traditional systemic chemotherapy, radiotherapy, endocrine therapy, and surgery, interventional therapy has been a focus of attention for clinical practitioners in LABC. Similar to solid tumors such as lung cancer and primary liver cancer, breast cancer and axillary lymph node metastases have rich blood supply^17^. This provides an anatomical basis for interventional therapy. Through digital subtraction angiography (DSA), we can observe that the main blood supply arteries for breast cancer are the internal mammary artery, the lateral thoracic artery, the subscapular artery, and the suprascapular artery. The distribution of blood supply arteries varies depending on the location of the breast tumor. In the experimental group of 30 patients, 9 had tumors located in the upper inner and lower inner quadrants, mainly supplied by the internal mammary artery, while 24 axillary lymph node metastases were supplied by the lateral thoracic artery and the subscapular artery.

Attempts to increase the dose–response rate by dose escalation have failed due to unacceptable toxic reactions. Most cytotoxic drugs have steep dose–response curves, so increasing drug exposure increases the response rate, which is a basic pharmacokinetic principle. Transarterial chemoembolization (TACE) delivers high-dose drugs directly into the tumor tissue via the tumor-feeding arteries, allowing for increased drug absorption by the tumor tissue. In theory, transarterial chemotherapy can increase local drug concentration in the tumor. Compared with systemic chemotherapy, transarterial chemotherapy (IACT) is superior in the induction treatment of locally advanced breast cancer. In the 1990s, some scholars attempted to use arterial infusion chemotherapy to treat LABC and achieved certain efficacy^18–20^. However, due to limitations in imaging technology and pharmacokinetic studies at that time, superselective catheterization was not performed, resulting in incorrect selection of blood supply arteries and short duration of high-concentration drug action, limiting the clinical application of this technique.

Anthracycline drugs exhibit severe local toxic reactions, which has restricted their use in intra-arterial infusion chemotherapy. In early trials, we attempted intra-arterial treatment with doxorubicin, but unfortunately, many patients developed intolerable severe skin reactions, leading us to discontinue this method. Despite our exploration of measures such as slow injection and the use of micro-pumps to reduce drug concentration, half of the patients still experienced unbearable side effects. While prolonging the injection time could potentially reduce local reactions, it would also increase other risks and discomfort. Later, we discovered that drug-eluting beads could adsorb medication and release it slowly over an extended period, which is different from our traditional embolization and arterial chemotherapy. As a novel treatment modality, drug-eluting beads were approved by the ethics committee because they could slow down the release rate of the drug and reduce local side effects.

Animal experiments have confirmed that after embolization with drug-eluting beads, local blood drug concentrations reach a peak within 3 days and remain at that level for 7–14 days^21^, which better matches the pharmacokinetics and tumor cell proliferation cycle characteristics of cytotoxic drugs. In addition to embolizing tumor-feeding arteries, CalliSpheres® drug-eluting microspheres can provide sustained and slow release of chemotherapy drugs locally. This embolization technique has been widely used in liver cancer, lung cancer, renal cancer, and cervical cancer^22^, confirming its safety and effectiveness. However, there are few reports on its use in breast cancer, with only a small number of studies confirming its efficacy in patients with locally advanced unresectable breast cancer^23^. However, these studies had small sample sizes and lacked a control group receiving systemic chemotherapy, making it difficult to demonstrate the superiority of this treatment modality.

**Figure 6:**
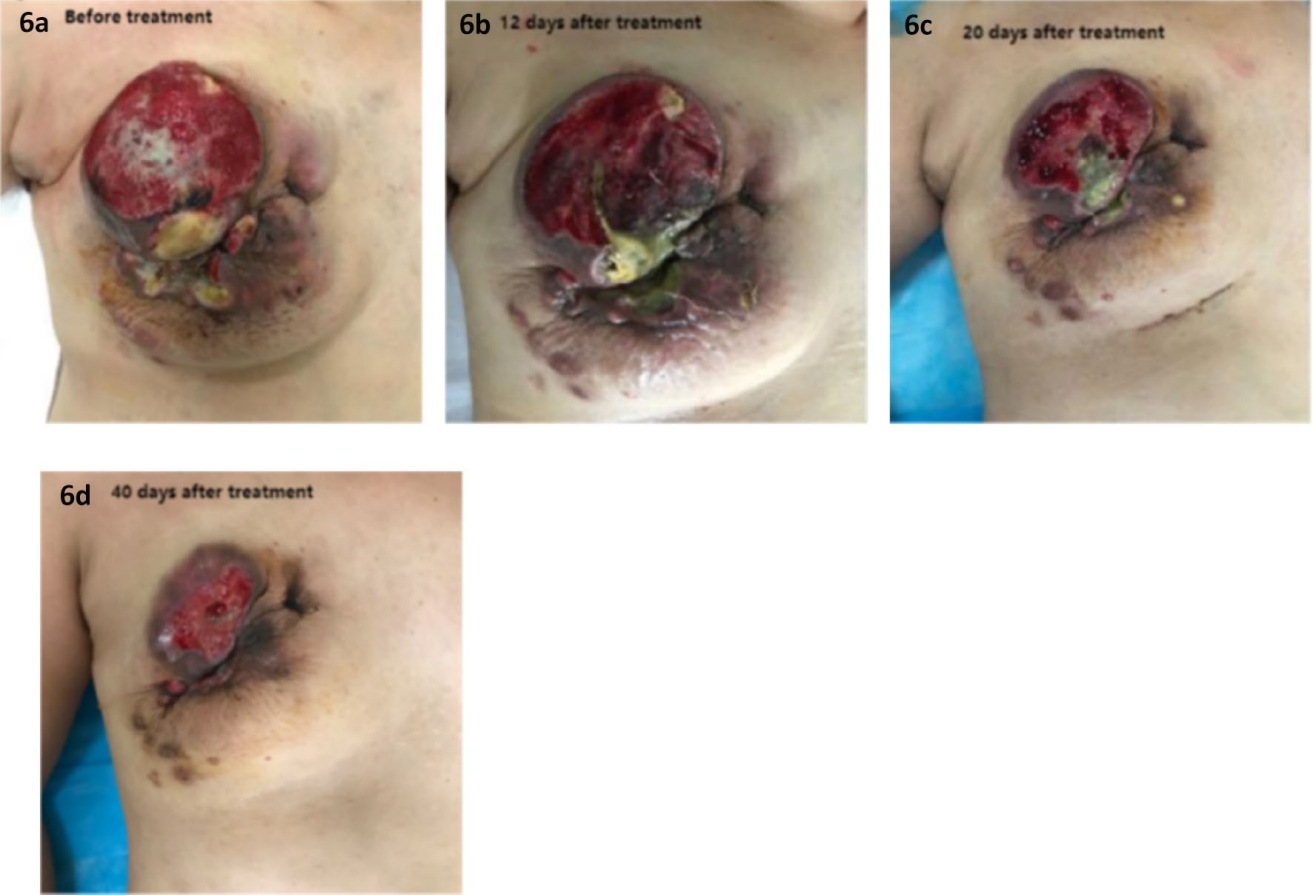
Changes after drug-loaded microsphere transcatheter arterial chemoembolization (DEB-TACE) in patients with breast cancer rupture and hemorrhage.

Since 1978, researchers have used arterial embolization to treat bleeding in locally advanced breast cancer patients. Initially, autologous blood clot and biological glue were used mainly for hemostasis. Later, gelatin sponge particles mixed with chemotherapy drugs were used for arterial embolization, resulting in significant tumor shrinkage^24–29^. We have used drug-eluting beads transarterial chemoembolization (DEB-TACE) in patients with ruptured bleeding breast cancer and achieved good results, with rapid shrinkage of breast and axillary tumors (Fig. 6). This suggests that this technique is a potentially feasible option for LABC patients.

However, there are some important considerations during the procedure: (1) Low-temperature protection measures should be taken for the skin tissue around the breast cancer and axillary lymph node metastases in the two hours before surgery. This helps to contract the microvasculature of normal tissues and avoid excessive entry of drug-eluting beads, which can lead to skin ulceration and necrosis. Low temperature also helps to alleviate skin pain. (2) Before deciding on the embolized vessel, cone-beam CT (CBCT) scanning should be performed and three-dimensional imaging reconstruction should be conducted to avoid incorrect perfusion chemotherapy and embolization. (3) Protective embolization using spring coils should be performed for arterial branches that do not supply blood to the tumor in order to reduce damage to the anterior abdominal wall (via the superior epigastric artery) and diaphragm (via the phrenic artery), thereby preventing abdominal skin ulcers and diaphragmatic nerve paralysis. (4) During the perfusion chemotherapy of axillary lymph node metastases, it is necessary to maintain pressure on the upper arm blood pressure cuff. This can help reduce the entry of albumin-bound paclitaxel into the normal vasculature of the upper limb. Although this drug has minimal damage to normal tissues, it can further increase the local drug concentration. (5) All embolizations must be performed using microcatheters for superselective embolization.

**Figure 7:**
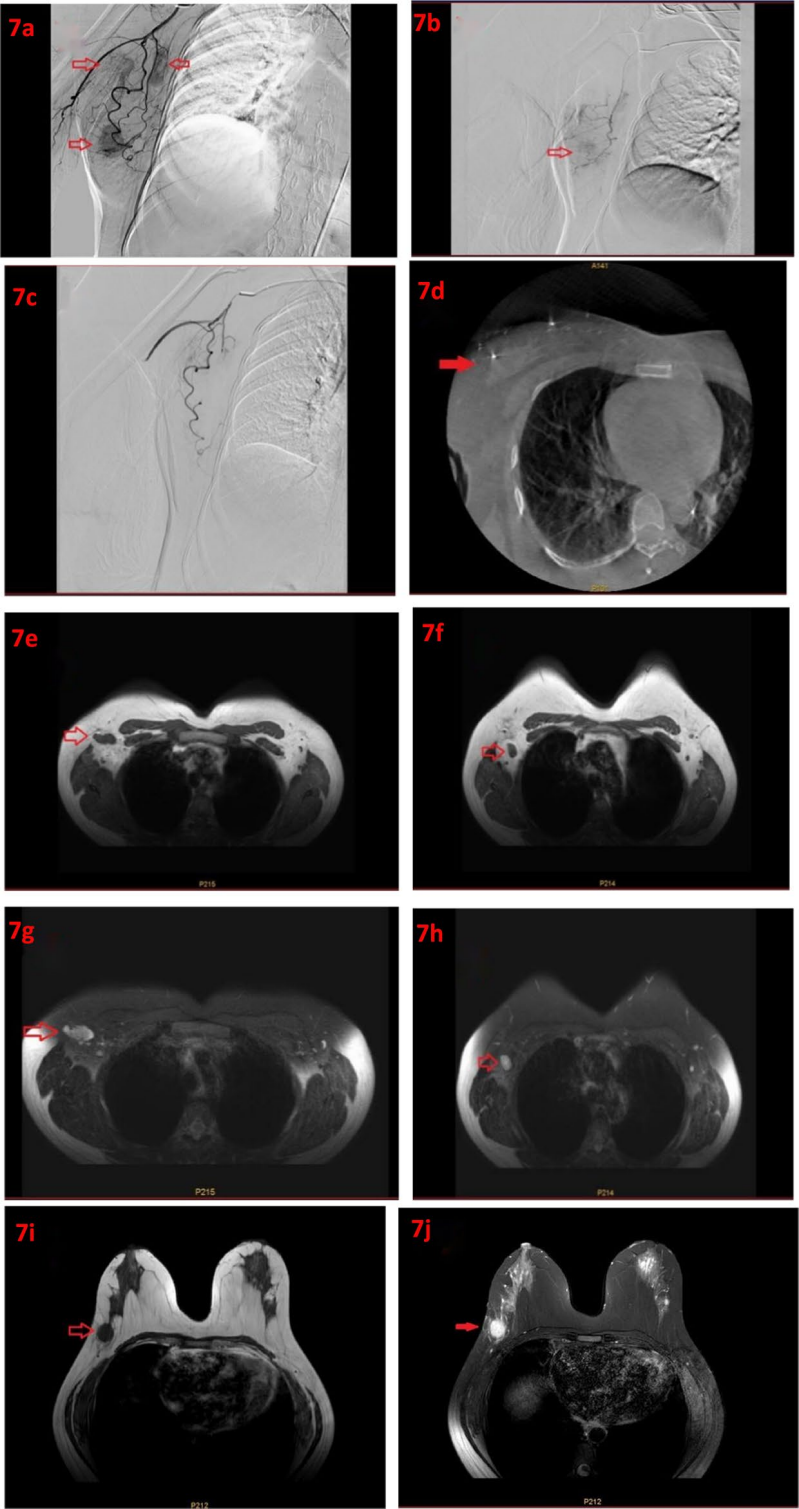
(**a**) Intraoperative imaging showing breast tumor and axillary lymph node staining. (**b**) Intraoperative imaging showing breast tumor and axillary lymph node staining. (**c**) Tumor staining disappeared after embolization. (**d**) Cone beam computed tomography (CBCT) showing breast tumor staining. (**e**) T1-weighted imaging of axillary lymph nodes (T1 WI). (**f**) T2-weighted imaging of axillary lymph nodes (T2 WI). (**g**) T1-weighted imaging (T1 WI). (**h**) T2-weighted imaging (T2 WI). (**i**) Axillary lymph nodes disappeared after treatment. (**j**) Primary breast lesions disappeared after treatment.

The number of patients achieving partial response (PR) after the first transarterial embolization in the experimental group was significantly higher than in the control group, and there was a statistically significant difference in overall response rate (ORR) between the two groups (*P* < 0.05), indicating that this technique had significant advantages over traditional systemic chemotherapy in rapidly shrinking tumor size. In terms of overall treatment efficacy, there were significantly more patients achieving complete response (CR) in the experimental group compared to the control group, and no patients in the experimental group experienced disease progression, whereas 3 patients in the control group had disease progression. The ORR comparison between the two groups had a *P* value of 0.02, and the pCR comparison had a *P* value of 0.005, which preliminarily demonstrated the superiority of the technique combining transarterial embolization with systemic chemotherapy (Fig. 7 shows the surgical procedure and the MRI images before and after surgery). Analyzing the reasons, the drug-eluting microsphere embolization we utilize differs from traditional embolization materials. These drug-eluting microspheres have the capacity to adsorb anthracycline drugs, which leads to a gradual release of the medication within the tumor. Without altering the drug dosage, this increases the concentration of the drug within the tumor tissue, offering a more advanced method of drug administration. There were no differences in the comparison of tumor regression between the two groups regarding primary lesions and metastatic lymph nodes, suggesting that the treatment approaches used in both the experimental and control groups were effective. Unfortunately, there was no significant difference in tumor regression of the primary breast lesion between the two groups (*P* = 0.051), but the experimental group showed significant superiority in terms of regression of axillary lymph node metastases (*P* = 0.003), which may be related to the richer blood supply in lymph node metastases. We are also trying this technique in HR−/HER2− breast cancer, and the initial results are even better, but the sample size is small, and further research will be published.

**Figure 8:**
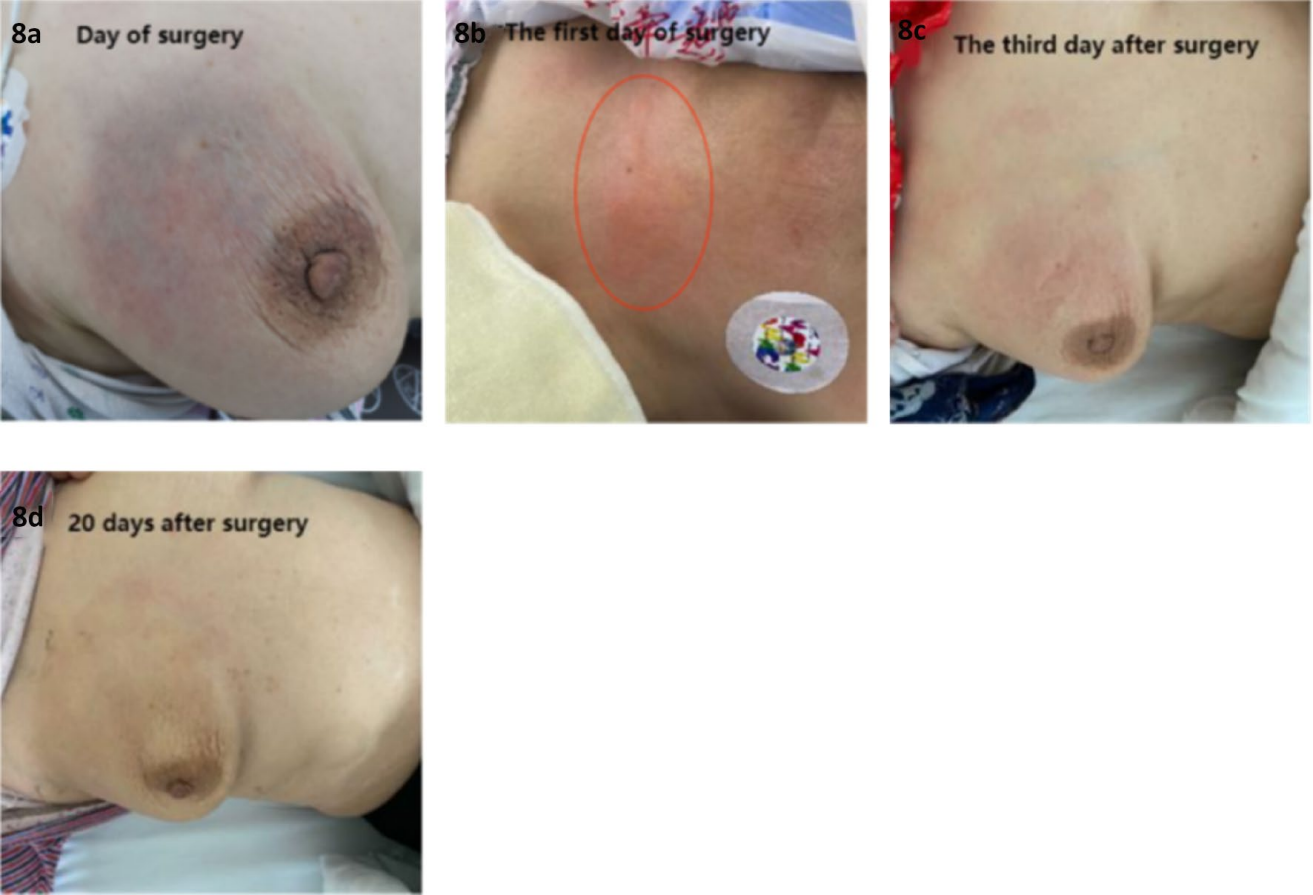
After surgery, different degrees of skin redness and blisters can subside spontaneously.

In terms of complications, one patient in the experimental group developed a local abscess. Retrospective imaging analysis revealed that the tumor invaded the central duct of the breast and the skin tissue around the nipple before surgery. After discharge, the patient developed blisters, which led to infection after treatment at a local clinic. This may be related to incomplete disinfection. Fortunately, the axillary lymph nodes were significantly reduced, and the patient underwent radical surgery after approximately 3 weeks postoperatively. In addition, 8 patients in the experimental group experienced varying degrees of redness, blisters, and pain after surgery. The blisters were absorbed on their own and skin pigmentation occurred afterwards (Fig. 8). We hypothesize that the cause of the skin redness might be related to the anthracycline drugs entering the interstitial tissues, due to the injection pressure and increased permeability of the vascular walls, but none of the patients had skin ulceration, this is different from the long-term skin ulceration observed after the extravasation of anthracycline drugs through intravenous injection. This further confirms the safety of this technique. Only one patient required opioid analgesia. There were no significant differences between the two groups in terms of white blood cell reduction, vomiting, creatine kinase-MB (CK-MB) levels, and brain natriuretic peptide (BNP) levels. All patients underwent surgical treatment after completing neoaAt the time of enrollment, we identified an anomaly in the proportion of ER−/PR+ cases. According to multiple studies, breast cancer patients with the ER−/PR+ phenotype may only represent a minority of all breast cancer patients, estimated to be around 1–4%, as most PR-positive tumors also express ER. We speculate that this could be related to the small size of the sample. Of course, it could also be associated with differences in population lifestyles, genetics, and diagnostic techniques, which is another limitation of this study. Djuvant therapy, and there were no delays in wound healing.

## The limitations of this study

This research is focused on patients who are undergoing neoadjuvant chemotherapy prior to surgery, with biopsy samples being obtained through core needle aspiration. This results in us only being able to collect a limited amount of tissue samples, which hinders our attempts at tumor grading. We have requested that pathologists grade these small samples, but they consider grading based on such limited samples to be both challenging and unreliable. In certain cases, patients who undergo surgical operations after treatment exhibit significant tumor necrosis, leaving behind only a minimal number of tumor cells, making it impossible to grade the tumors. This inability to grade tumors also represents a limitation of our study. This could lead to the inclusion of low-grade ER+/HER2− tumors in the cohort, thereby affecting the accuracy of the outcomes.

At the time of enrollment, we identified an anomaly in the proportion of ER−/PR+ cases. According to multiple studies, breast cancer patients with the ER−/PR+ phenotype may only represent a minority of all breast cancer patients, estimated to be around 1% to 4%, as most PR-positive tumors also express ER. We speculate that this could be related to the small size of the sample. Of course, it could also be associated with differences in population lifestyles, genetics, and diagnostic techniques, which is another limitation of this study.

We performed biopsies on patients who still showed tumor signals on MRI after treatment and who achieved complete remission according to the mRECIST criteria. Patients whose tumors had completely disappeared after treatment and had been confirmed by biopsy to have complete necrosis proceeded to complete neoadjuvant chemotherapy. In the experimental group, 7 patients underwent repeat biopsies, while in the control group, no patients received biopsies.

Due to family reasons, comorbidities, and physical fitness, 4 patients in the experimental group did not undergo surgical treatment. Their MRI scans still showed tumor signals, but according to the mRECIST criteria, they achieved complete remission. We carried out repeated biopsy punctures of the breast primary lesions and axillary lymph nodes, and the pathological results were negative, leading us to judge them as PCR (pathologic complete response). In the control group, 3 patients did not undergo surgery and did not achieve complete remission according to the mRECIST criteria, and were thus judged as PR (partial response). The rest of the patients underwent modified radical mastectomy for breast cancer after neoadjuvant therapy.It is noteworthy that another patient had a negative pathology report after repeated biopsy post-treatment, but a subsequent surgical excision of the tissue revealed a small amount of residual tumor cells. We considered the potential inconsistency between pathological complete response from a small-sample biopsy and that from a large tissue sample, but the extent of this discrepancy is still unclear. This is a limitation of small-sample tissue biopsies, but it definitely has an impact on the study results.This is a point that we necessarily need to clarify.

## Conclusion

In conclusion, based on our current observations, the combination of drug-eluting microspheres and transarterial chemoembolization (DEB-TACE) with systemic chemotherapy provides superior short-term treatment efficacy compared to traditional systemic chemotherapy for locally advanced HR+/HER2− breast cancer (LABC). Especially in terms of lymph node metastasis, the reduction is more pronounced. The overall safety assessment is reliable. We acknowledge the limitations of this study, including the small number of patients and the short follow-up period. The primary objective of this study was to explore a more effective novel adjuvant therapy to reduce tumor progression during neoadjuvant chemotherapy. It is noteworthy that no tumor progression was observed in the experimental group. However, whether the increase in pCR achieved by combining systemic chemotherapy with drug-eluting microsphere embolization can translate into long-term progression-free survival (PFS) and overall survival (OS) needs to be validated in multi-center, large-scale studies.

## Data availability

The data supporting this study can be obtained from Zhang Jingjun, MD and Shou Ffeng, MD.
